# Prolonged Post-Exercise Hypotension: Effects of Different Exercise Modalities and Training Statuses in Elderly Patients with Hypertension

**DOI:** 10.3390/ijerph18063229

**Published:** 2021-03-20

**Authors:** Ferdinando Iellamo, Giuseppe Caminiti, Matteo Montano, Vincenzo Manzi, Alessio Franchini, Annalisa Mancuso, Maurizio Volterrani

**Affiliations:** 1 Cardiology Rehabilitation Unit, S.Raffaele IRCCS, 00163 Rome, Italy; giuseppe.caminiti@sanraffaele.it (G.C.); mat.montano89@gmail.com (M.M.); franchini.fkt@gmail.com (A.F.); annalisamancuso76@gmail.com (A.M.); maurizio.volterrani@sanraffaele.it (M.V.); 2 Dipartimento di Scienze Cliniche e Medicina Traslazionale, Università Tor Vergata, 00133 Rome, Italy; vimanzi@yahoo.com

**Keywords:** post-exercise hypotension, exercise: exercise training, hypertension

## Abstract

Background: In this study, we aimed at comparing the effects of three different exercise modalities on post-exercise hypotension (PEH) in elderly hypertensive patients and at investigating whether PEH responses to the same exercises are affected by their training status. Methods: Thirty-six male sedentary hypertensive patients over 60 years old, were included. They were divided into three groups each one corresponding to a different exercise modality, i.e., aerobic continuous exercise (ACE), high-intensive interval exercise (HIIE), and combined (aerobic and resistance) exercise (CE). PEH was assessed in each group by ambulatory blood pressure monitoring (ABPM) in two different conditions as follows: (1) sedentary status and (2) trained status, at the end of a 12 week of ACE training program. A cardiopulmonary test was performed before and at the end of the training program. Results: In the sedentary status, 24-h and nocturnal systolic and diastolic blood pressure (BP) decreased in all groups as compared with top pre-exercise, with a greater but not significant reduction in the ACE and CE groups as compared with HIIE. ACE and HIIE groups presented a more sustained PEH than CE. In the trained status, 24-h and nighttime systolic and diastolic BP decreased significantly only after HIIE, but were unchanged as compared with pre-exercise in the ACE and CE groups. Conclusions: ACE and CE produced greater PEH than HIIE in sedentary elderly hypertensive patients. However, after training, HIIE produced the greater and more sustained PEH. The training status appears to exert significant effects on PEH produced by different exercise modalities.

## 1. Introduction

Exercise training is a well-established nonpharmacological treatment for patients with hypertension as well as other cardiovascular diseases. International guidelines strongly recommend exercise training, particularly aerobic continuous training (ACT), for primary and secondary prevention of hypertension with 1 A level of evidence [1,2]. Currently, training programs that are followed for the management of hypertensive patients often include high-intensity interval training (HIIT) and combined exercise (CE) that includes resistance training routines in addition to aerobic exercise. This exercise training modality is increasingly attractive because it might elicit several additional metabolic, hormonal, and muscular benefits in addition to blood pressure (BP) lowering [3,4,5]. Frequently, BP is reduced below resting pre-exercise levels even after a single exercise bout or exercise session, the so-called post-exercise hypotension (PEH). PEH is believed to be predictive of the chronic anti-hypertensive effect of exercise training programs [6]. PEH has been studied in both normotensive and hypertensive subjects with variable length observation periods after exercise [7,8,9]. In hypertensive patients, ambulatory blood pressure monitoring (ABPM) has the advantage to extend BP measurements over 24 h, thus, providing a more exhaustive evaluation of BP profile after exercise. ABPM provides information about the dynamics of BP during daily life and is now being increasingly recommended for both diagnostic and therapeutic purposes [10]. In addition, evidence is available that in populations, ABP values are more predictive of cardiovascular risk than office BP measurements [11].

Indeed, a single session of ACT has been reported to induce a sustained reduction in systolic and diastolic BP that is maintained up to 22 h after the session in hypertensives as compared with normotensives [12]. However, a recent review [13] reported that only 13% of studies on PEH used 24-h ABPM after exercise to assess prolonged BP-lowering effects of this therapeutic strategy. Hence, ABPM appears mandatory to firmly establish whether the effect of exercise on blood pressure is sustained over time (i.e., up to 24 h) or represents only a short-lasting post-effect of exercise. Assessing the duration of PEH effect could be of clinical relevance, considering the importance currently gained by ABPM [10,11]. Moreover, in the context of exercise therapy, to the best of our knowledge, there has been limited investigation regarding whether exercise modalities other than ACT are also capable of inducing PEH over a 24-h time period.

In addition, whether the training status affects PEH in hypertensive patients is also unknown. In healthy men, PEH was similar in both trained and sedentary subjects [14]. This aspect could be of clinical relevance because it could impact on exercise prescription, that is, maintaining or changing a given exercise prescription, in terms of type and intensity.

Accordingly, in this study, we aimed at comparing the effects of three different types of exercise modalities on 24-h PEH in elderly untrained hypertensives and at investigating whether the PEH response to the same types of exercise is dependent on their training status.

## 2. Methods

We enrolled 45 sedentary male patients with hypertension (lasting more than 1 year), under stable pharmacological treatment, referred by their physicians to a cardiac rehabilitation program. The following inclusion criteria were adopted: patients not enrolled in exercise training programs in the previous six months, age over 60 years, and clinical BP levels not exceeding 160/100 mmHg. The following exclusion criteria were adopted: secondary hypertension, significant heart valve diseases, hypertrophic cardiomyopathy, signs and/or symptoms of myocardial ischemia during an initial ergometric test, uncontrolled arrhythmia, neurological and or orthopedic conditions contraindicating or limiting exercises, significant chronic obstructive pulmonary disease (FEV1 < 50%) or peripheral arterial disease.

### 2.1. Study Design

**Figure 1:**
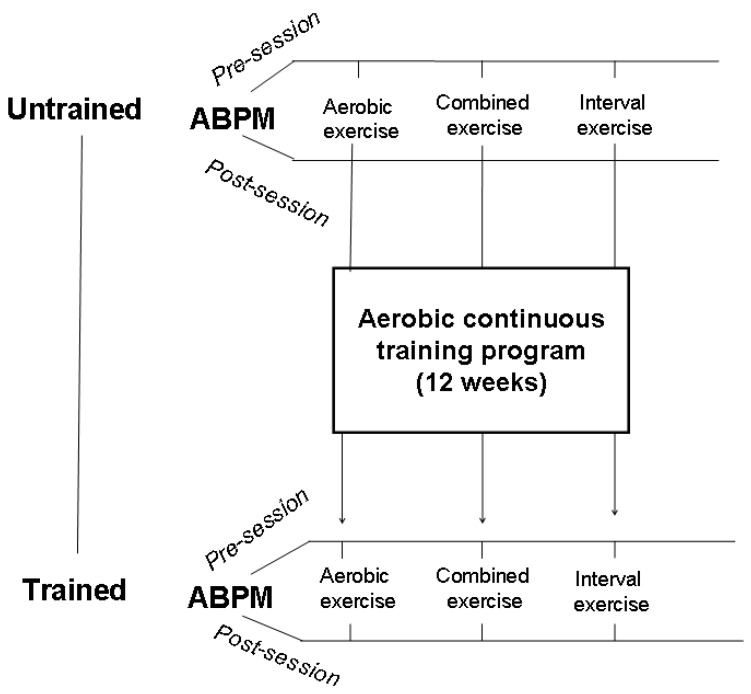
Flowchart of the study. ABPM, ambulatory blood pressure monitoring.

After a preliminary visit which included history collection and a physical examination, patients were randomly divided into three groups, each group underwent an acute session of different exercise modalities, i.e., aerobic continuous exercise (ACE), high-intensity interval exercise (HIIE), or combined exercise (CE) that consisted of aerobic + resistance exercises. All patients signed an informed consent to participate in the study that was approved by the local ethics committee (IRCCS San Raffaele, protocol no. 15/2018). Randomization was computer based and was provided internally and independently from the investigating physicians. As a baseline, all patients performed a cardiopulmonary test in order to rule out exercise-induced ischemia and other contraindications to exercise and to establish training intensity. PEH was evaluated by ABPM and it was assessed in each group under the following two different conditions: (a) before and after the single session of each exercise modality before starting the training program and (b) at the end of a 12-week aerobic continuous training (ACT) program, again after a single session of each exercise modality, in order to assess the effects of the training status (Figure 1). This latter was carried out within one week from the last session of the exercise training program. ABPMs always started within one hour after the end of the exercise session. The experimental exercise sessions were performed in the morning, between 8:30 and 10:00, and lasted 45 min. Patients were asked to not smoke, to have a light breakfast at least 2 h before the start of the session, and to regularly take their morning drugs.

ACE consisted of a 45 min walk on a treadmill at 55–70% of peak oxygen consumption (VO_2_). Patients, in the HIIE group, performed three peaks of high-intensity exercise, each one lasting 5 min at 80–95% of VO_2_ spaced by three intervals of lower-intensity exercise, each one lasting 10 min. Patients, in the CE group, walked for 25 min on a treadmill at 55–70% of peakVO_2_, and then they performed resistance exercises with arms and legs, consisting of 2 sets of 10 repetitions at 60% and 1 repetition at maximum (RM), with 2 min rest between sets. Resistance training consisted of the following exercises: leg press and extension, shoulder press, chest press, low row and vertical traction (Technogym Wellness System, Technogym, Cesena, Italy). Technogym’s ergometers employed in this study are specifically designed for different muscle groups and have the capability of detecting directly (and recording) the 1 RM. Endurance exercise was performed before resistance exercises, according to the priority training principle that exercises aiming to improve the most important parameters of performance should be performed first.

### 2.2. Training Program

Each exercise session of the training program included the following: 10 min of warm-up and cool-down and 40 min of aerobic exercise with the treadmill at 55–70% VO_2_ max. At baseline and at the end of the training program, all patients underwent a progressive incremental treadmill test until volitional fatigue with monitoring of gas exchange (Vmax29 C, SensorMedics, Yorba Linda, CA, USA) using a modified Bruce protocol. Peak VO_2_ was defined as the highest VO_2_ observed during exercise averaged for a 30 s recording time during the last minute. Respiratory exchange ratio was calculated at the same time point.

Twenty-four-hour ABPM was performed with a validated oscillometric device (BP one, LondonUK [15]. Recording was programmed to obtain BP readings at 15-min intervals from 6.00 a.m. to 10.00 p.m. and 20-min intervals from 10.00 p.m. to 6.00 a.m.

Data were accepted if at least 75% of the measurements were obtained successfully. On the day of the ABPM, patients were instructed to maintain their usual activities and medications. We distinguished the following three time periods: daytime (from 10:15 a.m. to 10:20 p.m.), nighttime (from 11 p.m. to 4:30 a.m.), and early morning (from 4:50 a.m. to 10:00 a.m.). The latter time period was to account for morning surges in BP which eventually occurring during this period.

### 2.3. Statistical Analysis

Data are expressed as mean ± SD. Changes in cardiopulmonary parameters were evaluated by paired *t*-test. Analysis of variance was used to compare the ABPM values among groups and sessions, followed by Tukey’s post hoc test. BP net differences (Δ pre-session to post-session) were compared by means of one-way analysis of variance, followed by Tukey’s post hoc test. The level of significance was set at *p* < 0.05. Data were analyzed using SPSS 14.0 software.

## 3. Results

Forty-five patients were selected for the study. Four patients dropped out during the training program, because they were unwilling to continue the study. Two patients dropped out for medical reasons (knee injury in one case and low-back pain during exercise training in the other). Three patients were excluded from analyses due to protocol violation in terms of changes in the antihypertensive medication. Therefore, thirty-six out of 45 enrolled patients completed the study. The subjects’ characteristics are reported in Table 1. Patients had a high compliance to the exercise training protocol (attended sessions/planned sessions × 100 = 95.2%).

**Figure 2:**
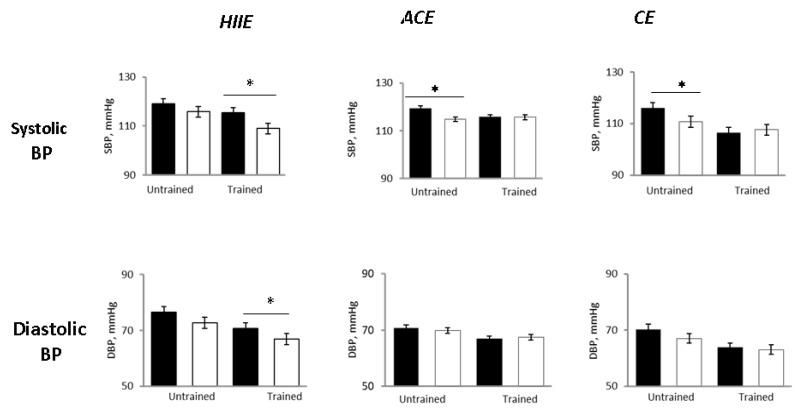
Post-exercise changes in ABPM 24-h systolic and diastolic blood pressure (BP) in the three study groups before (black bars) and after (white bars) a single exercise session in the untrained and trained status. HIIE, high intensity interval exercise; ACE, aerobic continuous exercise; CE, combined exercises. * *p* < 0.05 vs. pre exercise.

**Figure 3:**
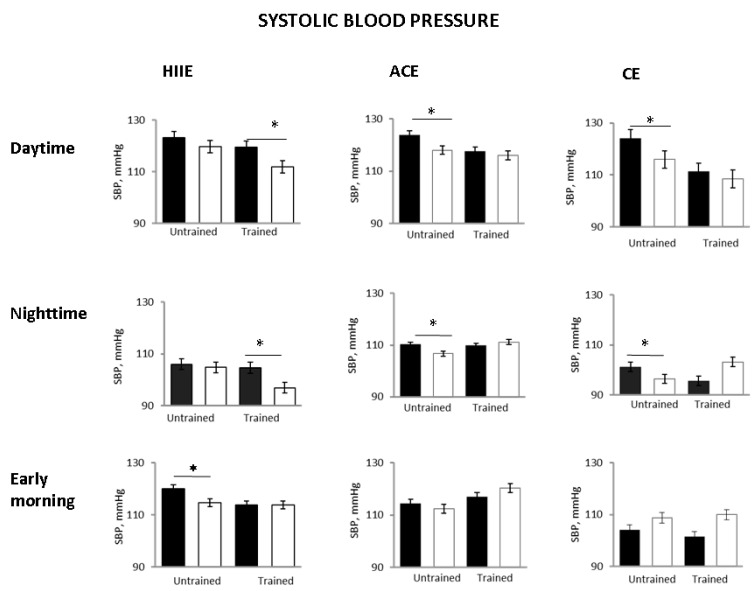
Post-exercise changes in ABPM systolic BP in the three study groups in the different 24-h periods before (black bars) and after (white bars) a single exercise session in the untrained and trained status. HIIE, high intensity interval exercise, ACE, aerobic continuous exercise; CE, combined exercises * *p* < 0.05 vs. pre exercise.

In the untrained status, 24-h SBP decreased in all groups as compared with pre-exercise, with the BP lowering being significant in the ACE and CE groups but not in HIIE (Figure 2). The 24-h DBP decreased to a similar extent in all groups, although not significantly (Figure 2). Daytime and nighttime SBP were significantly reduced in ACE and CE, but, again, not in HIIE. Early morning SBP decreased significantly only in the HHIE group (Figure 3). In all three groups, exercise sessions did not significantly affect HR (Table 1).

No adverse events occurred during the different exercise sessions.

At the end of the training program, peak VO_2_ was found to be significantly increased (from 21.5 ± 2.7 to 24.3 ± 4.1, mL/min/Kg, *p* = 0.004) as it was RER at peak exercise (from 1.14 ± 0.9 to 1.22 ± 0.7, *p* = 0.002). The duration of the ergometric test was also significantly increased (from 366.4 ± 55 s to 448.3 ± 72 s, *p* = 0.0001) while resting HR decreased from 64.4 ± 15.4 to 61.2 ± 18.6 b/min, *p* = 0.02).

In the trained status (i.e., after 12-weeks training program) pre-exercise values of 24-h systolic and diastolic BP were significantly lower as compared with pre-exercise values in the untrained status (Figure 2), confirming the effectiveness of training on lowering BP (in addition to improving functional capacity).

However, in the trained status after the single exercise sessions, 24-h SBP and DBP did not change significantly as compared with pre-exercise in the ACE and CE groups, whereas they were significantly reduced in the HIIE group (Figure 3). Daytime SBP was also significantly lower in the HIIE group but not in the ACE and CE groups. Nighttime SBP showed a significant decrease in the HIIE group while it was not significantly affected in the other groups (Figure 3). In other words, in the trained status, only HIIE was capable of reducing whole day and time periods related to BP.

**Figure 4:**
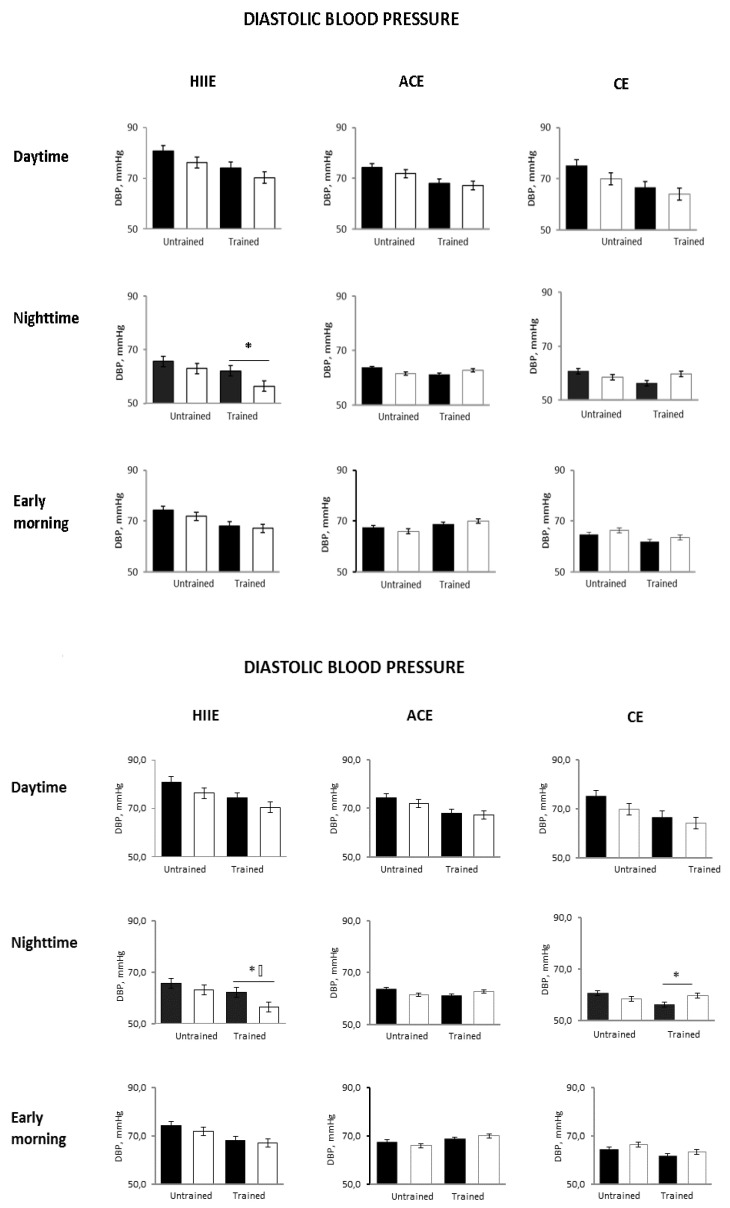
Post-exercise changes in ABPM diastolic BP in the three study groups in the different 24-h periods before (black bars) and after (white bars) a single exercise session in the untrained and trained status. HIIE, high intensity interval exercise; ACE, aerobic continuous exercise; CE, combined exercises * *p* < 0.05 vs. pre exercise.

Compared with the untrained status, early morning SBP was unchanged in all groups. The 24-h and nighttime diastolic BP decreased only in the HIIE group (*p* = 0.04 and *p* = 0.0001 between groups, respectively) (Figure 2 and Figure 4).

## 4. Discussion

The main findings of this study are that, in untrained hypertensive patients, PEH does occur to a similar extent over 24 h after a single exercise session of any modality, whereas after a prolonged period of currently recommended aerobic endurance training, PEH after a single exercise session does occur only in response to HIIE. Hence, the training status can influence PEH.

In the untrained status, the exercise-stress stimulus induced a prolonged decrease in BP, regardless of the mode of exercise administered, that is, ACE, HIIE, or CE.

ACE and HIIE presented a more sustained PEH as compared with CE, with systolic and diastolic BP reductions still evident in the early-morning phase. Several studies involving aerobic exercise have reported reductions in systolic and diastolic BP levels sustained over several hours [8,9]. In elderly patients with hypertension, Ferrari et al. [16], using ABPM, observed that sessions of CE produced PEH similar to that occurring with ACE, although less sustained. The PEH effect of HIIE has been poorly studied in elderly hypertensive patients utilizing ABPM. In a study performed in middle-aged sedentary hypertensives, Ciolac et al. [17] demonstrated a similar reduction of SBP and DBP after a single session of both ACE and HIIE, with BP reductions sustained over almost the entire 24-h registration period. Similar results have been reported by Edwards et al. [18] but in healthy young individuals. Conversely, two studies showed a greater post-exercise reduction of SBP after a single session of HIIE as compared with ACE [19,20]. However, these studies did not use ABPM and their observations were limited to the first 60 min post exercise. We are not aware of direct comparisons between HIIE and CE on PEH in hypertensive populations. Therefore, additional data are required regarding the effects of different kinds of exercise on 24-h PEH in hypertensive patients. We found significant reductions in daytime and nighttime SBP in response to ACE and CE, whereas DBP was substantially unaffected by exercises. These reductions in nighttime SBP could be of potential clinical relevance inasmuch as nighttime SBP represents a significant risk factor. Interestingly, early morning SBP, which is a time of surges in BP, was also reduced but this decrease was significant only in the HIIE group, thus, limiting the implications of such finding. This is the first study in hypertensive patients to compare the PEH by ABPM before and after single sessions of exercises that comprise the most employed exercise modalities. We cannot comment on the mechanism(s) of exercise-induced PEH, as this was not investigated as part of the objectives of this study. However, an earlier study from this lab [7] in a similar hypertensive population, indicated that PEH is mediated mainly by a peripheral vasodilation, which may involve metabolic factors linked to post-exercise hyperemia. This vasodilator effect appeared to override a concomitant reflex sympathetic activation directed to the vasculature, with the possible aim of countering excessive decreases in BP [7]. In that study [7], no changes in cardiac output were observed post exercise, whereas in two patients in which PEH did not occur, we observed a lack of changes in peripheral resistances.

A strength of the present study is that we evaluated the effects of a single session of different exercise modalities on PEH and also investigated whether these effects would persist after a long period of currently recommended training.

### PEH in the Trained Conditions

The PEH induced by the three exercise modalities differed significantly when the experimental protocol was repeated at the end of the exercise training program. In particular, we observed that HIIE determined the more intense and sustained PEH response as compared with both ACE and CE. Remarkably, the BP reduction produced by HIIE was still observed during the night, at the time when SBP and DBP dropped significantly as compared with pre-exercise values. Conversely, in the ACE and CE groups, daytime and nighttime SBP and DBP did not change as compared with pre-exercise conditions. Our data seem to indicate that the training status significantly impacts the PEH produced by single sessions of different exercise modalities. The influences of training status on PEH in elderly hypertensive subjects have been poorly studied. The blunted BP response observed in our study could be related to the attenuation of BP benefits described during long-term non-pharmacological programs by Hinderliter et al. [21]. Our findings, potentially, could also be due to a lower threshold of BP response, where it cannot be decreased further below a given level in order to prevent hypotension [22]. Interestingly, HIIE still maintained its capability to also induce SBP lowering in the trained state. A plausible explanation for this finding is that in the trained status, in which several adaptations have occurred as a result of training, a stronger stimulus, such as that produced by the repeated increase in shear stress on the endothelial function by interval exercises [23,24], is needed to improve further vasodilation and, in turn, reduce BP.

This speculation could be physiologically interesting (and testable), but what is really clinically important is the capability of all types of physical activity to produce a significant reduction in BP lasting up to 24 h, more consistently for moderate continuous-type activity. The observation in this study of similar BP values in the ABPM recorded after the single session exercises in the untrained state with those recorded in the ABPM before the single session exercises in the trained state (Figure 2 and Figure 3) would support this suggestion. At variance with our results, Imazu et al. [25] reported that trained hypertensive patients presented lower values of SBP and DBP than the sedentary participants, after a single bout of aerobic continuous exercise, with this effect lasting for a period of 12 h. Differences between our study and that of [25] could be related mainly to a different methodological approach and, in particular, to the study design (cross-over versus longitudinal). Alternatively, it could be hypothesized that post-exercise hypotension is blunted, or even absent, in trained, hypertensive individuals under pharmacological treatment [14], unless a stronger stimulus (e.g., bouts of HIIE) is applied.

In the light of our results, we hypothesize the opportunity to periodically reassess the PEH response of elderly hypertensive patients undergoing long-term exercise training programs, in order to verify their responsiveness to a specific training program and, possibly, to optimize the exercise intensity or change the exercise modality, with the goal of obtaining the best possible anti-hypertensive effect.

There were some limitations in our study. This study included only male hypertensives under pharmacological therapy, and hence our results cannot be generalized to female and/or untreated patients. Moreover, we employed only the aerobic continuous exercise modality to train our hypertensive patients. This choice was dictated by current guidelines that recommend ACE as the preferred training modality in hypertension [26,27], until more efficacy and safety data of other exercise training modalities are available. Therefore, it is possible that different training modalities could produce, in time, different results. Finally, we cannot comment on the mechanisms of blood pressure lowering effects of ACE, as This was beyond the aim of our study. Endurance exercise training has the potential to have an impact on several mechanisms involved in BP regulation which include changes in vasodilator capacity [28,29] autonomic nervous system functioning [7,30], humoral substances, and their interplay.

## 5. Conclusions

In conclusion, we observed that in untrained elderly hypertensive subjects, ACE and CE produced greater 24-h PEH effects than HIIE. Conversely, in the trained status, the 24-h PEH response to ACE and CE tended to be blunted, while the 24-h PEH response to HIIE was amplified as compared with the untrained condition. Periodic assessment of 24-h PEH in the course of long-term training programs could prove to be helpful for optimizing training prescription, in terms of exercise modality and intensity and obtain optimal anti-hypertensive effect.

## Figures and Tables

**Table 1:** Baseline characteristics of patients.

	HIIE (*n* = 12)	ACE (*n* = 12)	CE (*n* = 12)
Age, y	64.5 ± 7.2	66 ± 4.7	65.5 ± 3.8
BMI, kg/m^2^	28.9 ± 1.7	27.9 ± 4.3	27.3 ± 3.8
Diabetes, *n* (%)	2 (16)	3 (25)	2 (16)
Dislipidemia, *n* (%)	5 (42)	6 (50)	6 (50)
Smoking habit, *n* (%)	5 (42)	7 (58)	4 (33)
Resting SBP, mmHg	122.6 ± 28.4	121.8 ± 33.1	121.2 ± 31.6
Resting DBP, mmHg	81.7 ± 16.4	81.3 ± 14.6	82.0 ± 11.3
Resting heart rate, bpm	62.2 ± 11.5	63.7 ± 8.3	62.4 ± 10.1
Coronary artery disease, *n* (%)	4 (33)	3 (25)	4 (33)
Carotid artery disease, *n* (%)	2 (16)	2 (16)	3 (25)
eGFR, mL/min	71 ± 13	68 ± 18	74 ± 14
N° of anti-hypertensive drugs	3.3 ± 0.8	3.1 ± 1.1	3.0 ± 0.7
Anti-hypertensive Treatment			
ACE-i/ARBs, *n* (%)	8 (67)	10 (83)	9 (75)
CCAs, *n* (%)	5 (42)	4 (33)	6 (50)
Betablockers, *n* (%)	5 (42)	5 (42)	3 (25)
Diuretics, *n* (%)	6 (50)	7 (58)	8 (67)
Other Drugs			
Antiplatelets, *n* (%)	4 (57)	5 (71)	5 (71)
Statins, *n* (%)	6 (50)	7 (58)	7 (58)
Ivabradine, *n* (%)	3 (25)	2 (16)	2 (16)
Ranolazine, *n* (%)	1(8)	2 (16)	-

HIIE, high intensity interval exercise; ACE, aerobic continuous exercise; CE, combined (aerobic + resistance) exercise; BMI, body mass index; SBP, systolic blood pressure; DBP, diastolic blood pressure; eGFR, estimate glomerular filtration rate; ACEi, angiotensin converting enzyme inhibitors; ARBs, angiotensin receptors blockers; CCA, calcium antagonists. No significant differences in any baseline characteristic were detected between the three exercising groups.

## Data Availability

The data presented in this study are available on request from the corresponding author.

## References

[1] Whelton P.K. (2018). 2017 ACC/AHA/AAPA/ABC/ACPM/AGS/APhA/ASH/ASPC/NMA/PCNA. Guideline for the prevention, detection, evaluation, and management of high blood pressure in adults. JACC.

[2] Williams B., Mancia G., Spiering W., Agabiti Rosei E., Azizi M., Burnier M., Clement D.L., Coca A., De Simone G., Dominiczak A. (2018). 2018 ESC/ESH Guidelines for the management of arterial hypertension. Eur. Heart J..

[3] Karstoft K., Winding K., Knudsen S.H., Nielsen J.S., Thomsen C., Pedersen B.K., Solomon T.P. (2013). The effects of free-living interval-walking training on glycemic control, body composition, and physical fitness in type 2 diabetic patients: A randomized, controlled trial. Diabetes Care.

[4] Caminiti G., Iellamo F., Manzi V., Fossati C., Cioffi V., Punzo N., Murugesan J., Volterrani M., Rosano G. (2014). Anabolic hormonal response to different exercise training intensities in men with chronic heart failure. Int. J. Cardiol..

[5] Lima L.G., Bonardi J., Campos G.O., Bertani R.F., Scher L.M., Moriguti J.C., Ferriolli E., Lima N.K. (2017). Combined aerobic and resistance training: Are there additional benefits for older hypertensive adults?. Clinics.

[6] Luttrell M.J., Halliwill J.R. (2015). Recovery from exercise: Vulnerable state, window of opportunity, or crystal ball?. Front. Physiol..

[7] Legramante J.M., Galante A., Massaro M., Attanasio A., Raimondi G., Pigozzi F., Iellamo F. (2002). Hemodynamic and autonomic correlates of postexercise hypotension in patients with mild hypertension. Am. J. Physiol. Regul. Integr. Comp. Physiol..

[8] Gomes Anunciação P., Polito M.D. (2011). A review on post-exercise hypotension in hypertensive individuals. Arquivos Brasil Cardiol..

[9] Cardoso C.G., Gomides R.S., Queiroz A.C., Pinto L.G., Lobo F.D., Tinucci T., Mion D., Forjaz C.L. (2010). Acute and chronic effects of aerobic and resistance exercise on ambulatory blood pressure. Clinics.

[10] Mallion J.M., Baguet J.P., Mancia G. (2006). European Society of Hypertension Scientific Newsletter: Clinical value of ambulatory blood pressure monitoring. J. Hypertens..

[11] Dolan E., Stanton A., Thijs L., Hinedi K., Atkins N., McClory S., Hond E.D., McCormack P., Staessen J.A., O’Brien E. (2005). Superiority of ambulatory over clinic blood pressure measurement in predicting mortality. The Dublin outcome study. Hypertension.

[12] Brandão Rondon M.U., Alves M.J., Braga A.M., Teixeira O.T., Barretto A.C., Krieger E.M., Negrão C.E. (2002). Postexercise blood pressure reduction in elderly hypertensive patients. J. Am. Coll. Cardiol..

[13] De Brito L.C., Fecchio R.Y., Peçanha T., Lima A., Halliwill J., Forjaz C.L.M. (2019). Recommendations in Post-exercise Hypotension: Concerns, Best Practices and Interpretation. Int. J. Sports Med..

[14] Senitko A.N., Charkoudian N., Halliwill J.R. (2002). Influence of endurance exercise training status and gender on postexercise hypotension. J. Appl. Physiol..

[15] Friz H.P., Sega R., Facchetti R., Primitz L., Beltrame L., Bombelli M. (2008). Accuracy evaluation of the ‘Cardiette BP one’ ambulatory blood pressure monitor. Blood Press. Monit..

[16] Ferrari R., Umpierre D., Vogel G., Vieira P.J.C., Porto Santos L., Bandeira de Mello R., Tanaka H., Fuchs S.C. (2017). Effects of concurrent and aerobic exercises on postexercise hypotension in elderly hypertensive men. Exp. Gerontol..

[17] Ciolac E.G., Guimaraes G.V., D’Avila V.M., Bortolotto L.A., Doria E.L., Bocchi E.A. (2009). Acute effects of continuous and interval aerobic exercise on 24-h ambulatory blood pressure in long-term treated hypertensive patients. Int. J. Cardiol..

[18] Edwards J.J., Taylor K.A., Cottam C., Jalaludeen N., Coleman D.A., Jonathan DWiles J.D., Rajan Sharma R., O’Driscoll J.M. (2021). Ambulatory blood pressure adaptations to high-intensity interval training: A randomized controlled study. J. Hypertens..

[19] Pimenta F.C., Montrezol F.T., Dourado V.Z., da Silva L.F., Borba G.A., de Oliveira Vieira W., Medeiros A. (2019). High-intensity interval exercise promotes post-exercise hypotension of greater magnitude compared to moderate-intensity continuous exercise. Eur. J. Appl. Physiol..

[20] Dos Santos J.M., Gouveia M.C., De Souza F.A., da Silva Rodrigues C.E., Santos J.M., Santos de Oliveira A.J., Oliveira Marques A.C., Barbosa B.T., Sales Suassuna J.A. (2018). Effect of a High-Intensity Interval Training Session on Post-Exercise Hypotension and Autonomic Cardiac Activity in Hypertensive Ederly Subjects. J. Exerc. Physiol..

[21] Hinderliter A.L., Sherwood A., Craighead L.W., Lin P.H., Watkins L., Babyak M.A., Blumenthal J.A. (2014). The Long-Term Effects of Lifestyle Change on Blood Pressure: One-Year Follow-Up of the ENCORE Study. Am. J. Hypertens..

[22] Ramirez-Jimenez M., Morales-Palomo F., Pallares J.G., Mora-Rodriguez R., Ortega J.F. (2017). Ambulatory blood pressure response to a bout ofHIIT in metabolic syndrome patients. Eur. J. Appl. Physiol..

[23] Cornelissen V.A., Smart N.A. (2013). Exercise training for blood pressure: A systematic review and meta-analysis. J. Am. Heart Assoc..

[24] MacDonald H.V., Pescatello L.S., Kokkinos P., Narayan P. (2019). Exercise and blood pressure control in hypertension. Cardiorespiratory Fitness in Cardiometabolic Diseases: Prevention and Management in Clinical Practice.

[25] Imazu A., Goessler K., Casonatto J., Polito M. (2017). The influence of physical training status on postexercise hypotension in patients with hypertension: A cross-sectional study. Blood Press Monit..

[26] Pescatello L.S., MacDonald H.V., Lamberti L., Johnson B.T. (2015). Exercise for Hypertension: A Prescription Update Integrating Existing Recommandations with Emerging Researh. Curr. Hypertens. Rep..

[27] Piepoli M.F., Hoes A.W., Agewall S., Albus C., Brotons C., Catapano A.L., Cooney M.T., Corrà U., Cosyns B., Deaton C. (2016). 2016 European guidelines on cardiovascular disease prevention in clinical practice: The sixth joint task force of the European Society of Cardiology and other societies on cardiovascular disease prevention in clinical practice. Eur. Heart J..

[28] Snell P.G., Martin W.H., Buckey J.C., Blomquist C.G. (1987). Maximal vascular leg conductance in trained and untrained men. J. Appl. Physiol..

[29] Halliwill J.R., Minson C.T., Joyner M.J. (2000). Effect of systemic nitric oxide synthase inhibition on postexercise hypotension in humans. J. Appl. Physiol..

[30] Narkiewicz K., Somers V.K. (1997). Endurance training in mild hypertension—Effects on ambulatory blood pressure and neural circulatory control. Blood Press Monit..

